# IRE1α inhibition attenuates neuronal pyroptosis via miR-125/NLRP1 pathway in a neonatal hypoxic-ischemic encephalopathy rat model

**DOI:** 10.1186/s12974-020-01796-3

**Published:** 2020-05-06

**Authors:** Juan Huang, Weitian Lu, Desislava Met Doycheva, Marcin Gamdzyk, Xiao Hu, Rui Liu, John H. Zhang, Jiping Tang

**Affiliations:** 1 Institute of Neuroscience, Chongqing Medical University, Chongqing, 400016 China; 2 Department of Physiology and Pharmacology, Loma Linda University, Risley Hall, 11041 Campus St, Loma Linda, CA 92350 USA; 3 Department of Neurology, Guizhou Provincial People’s Hospital, Guiyang, 550002 China; 4 Department of Anesthesiology, Loma Linda University, Loma Linda, CA 92350 USA; 5 Department of Neurosurgery, Loma Linda University, Loma Linda, CA 92350 USA

**Keywords:** Hypoxic-ischemic encephalopathy, IRE1α, miR-125, NLPR1, Inflammasome, Pyroptosis

## Abstract

**Background:**

Inhibition of inositol-requiring enzyme-1 alpha (IRE1α), one of the sensor signaling proteins associated with endoplasmic reticulum (ER) stress, has been shown to alleviate brain injury and improve neurological behavior in a neonatal hypoxic-ischemic encephalopathy (HIE) rat model. However, there is no information about the role of IRE1α inhibitor as well as its molecular mechanisms in preventing neuronal pyroptosis induced by NLRP1 (NOD-, LRR- and pyrin domain-containing 1) inflammasome. In the present study, we hypothesized that IRE1α can degrade microRNA-125-b-2-3p (miR-125-b-2-3p) and activate NLRP1/caspased-1 pathway, and subsequently promote neuronal pyroptosis in HIE rat model.

**Methods:**

Ten-day old unsexed rat pups were subjected to hypoxia-ischemia (HI) injury, and the inhibitor of IRE1α, STF083010, was administered intranasally at 1 h after HI induction. AntimiR-125 or NLRP1 activation CRISPR was administered by intracerebroventricular (i.c.v) injection at 24 h before HI induction. Immunofluorescence staining, western blot analysis, reverse transcription quantitative real-time polymerase chain reaction (RT-qPCR), brain infarct volume measurement, neurological function tests, and Fluoro-Jade C staining were performed.

**Results:**

Endogenous phosphorylated IRE1α (p-IRE1α), NLRP1, cleaved caspase-1, interleukin-1β (IL-1β), and interleukin-18 (IL-18) were increased and miR-125-b-2-3p was decreased in HIE rat model. STF083010 administration significantly upregulated the expression of miR-125-b-2-3p, reduced the infarct volume, improved neurobehavioral outcomes and downregulated the protein expression of NLRP1, cleaved caspase-1, IL-1β and IL-18. The protective effects of STF083010 were reversed by antimiR-125 or NLRP1 activation CRISPR.

**Conclusions:**

IRE1α inhibitor, STF083010, reduced neuronal pyroptosis at least in part via miR-125/NLRP1/caspase-1 signaling pathway after HI.

**Graphical Abstract:**

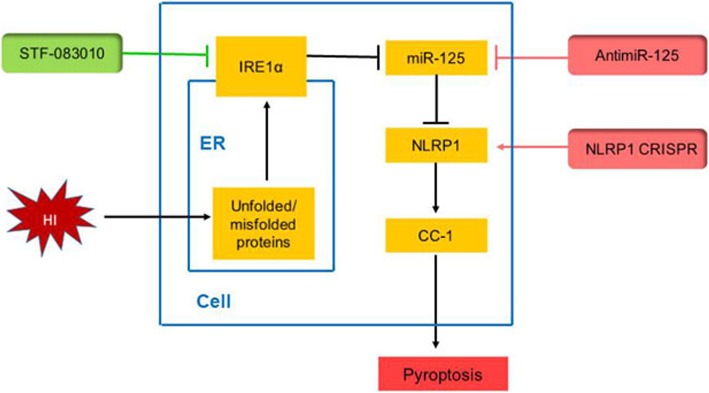

## Background

Neonatal hypoxic ischemic encephalopathy (HIE) is a devastating disease that causes perinatal brain injury and leads to prolonged neurodevelopmental consequences in infants including cerebral palsy, cognitive deficits, mental retardation, seizures, epilepsy, and other neurological disabilities [1–3]. Although there has been tremendous progress in understanding HIE pathologies, there is still a need to explore its pathophysiology and treatment modalities [3].

Growing evidence reveal that endoplasmic reticulum (ER) stress is involved in the pathogenesis of HIE [4–7]. ER stress is the condition where ER homeostasis is disturbed and the unfolded or misfolded proteins accumulate in the ER lumen, which can be caused by many physiological or pathological factors, such as hypoxia, ischemia, or changes in intracellular pH [5, 8–10]. Inositol-requiring enzyme 1 alpha (IRE1α), an ER transmembrane kinase-endoribonuclease (RNase), is a stress sensor receptor, which is activated by the accumulation of unfolded or misfolded proteins [11, 12]. It has been reported that sustained IRE1α activation causes rapid decay of some microRNAs (miRNAs), such as miR-17 and miR-125 [13].

miRNAs are a class of noncoding short single-stranded RNAs (~ 22 nt in length) that play a role in downregulating gene expression at the post transcriptional level via binding to complementary nucleotide sequences of the target mRNA [14–18]. Our previous research showed that IRE1α inhibition alleviated brain injury and restrained the activation of TXNIP/NLRP3 inflammasome via upregulating miR-17 expression level in neonatal HIE rat model [5]. However, whether IRE1 inhibition may be protective via regulation of miR-125 expression levels has not been studied.

Neuronal pyroptosis, a form of programmed neuronal cell death, initiated by caspase-1, is one of the important causes of neurological damage [19]. The nucleotide-binding oligomerization domain (NOD)-like receptor (NLR) pyrin domain-containing (NLRP) inflammasomes have recently been identified and shown to contribute to cell pyroptosis [20, 21]. Inflammasomes are the multi-protein complexes composed of a cytosolic pattern-recognition receptor, the enzyme caspase 1 and an adaptor protein that facilitates the interaction between the former two [22, 23]. Activation and homo-oligomerization of NLRP receptor induces the formation of NLRP inflammasomes, which convert precursor caspase-1 into cleaved caspase-1. The cleaved caspase-1 mediates the inflammatory responses including cleavage and secretion of inflammatory cytokines interleukin-1β (IL-1β) and interleukin-18 (IL-18), and then initiates the inflammatory form of cell death, referred to as pyroptosis [20, 24]. Up to now NLRP1 and NLRP3 inflammasomes are the most extensively studied inflammasomes [22, 25, 26]. An online search using a miRNA target prediction software, TargetScan (http://www.targetscan.org/), revealed that miR-125b-2-3p contains nucleotide sequences complementary to the highly conserved seed sequences in the NLRP1 mRNA 3’-UTR (Fig. 7). There are also several previous reports on miR-125 as a regulator of NLRP1 mRNA stability [27, 28]. Therefore, we deduce that IRE1 could at least partially regulate the expression level of NLRP1 via miR-125. We hypothesized that IRE1α inhibition will prevent neuronal pyroptosis via miR-125/NLRP1 pathway in the neonatal HIE rat model.

## Material and methods

### Animals

All experiments performed in this study were in compliance with the National Institutes of Health guidelines for the handling of laboratory animals and approved by Loma Linda University Institutional Animal Care and Use Committee. Sprague-Dawley rat mothers, with litters of 12 pups, were purchased from Envigo (Livermore, CA) and housed in regular light/dark cycle environment with humidity and temperature controlled. Post-natal day 10 (P10) pups, weighing 14–20 g, underwent hypoxia-ischemia surgery. A total of 142 unsexed rat pups were used in this study. Among them, 14 pups were excluded due to death during ischemia surgery or hypoxia induction.

### HIE rat model

The modified Rice-Vannucci method was performed to create the HIE rat model as previously described [29, 30]. Briefly, rat pups were anesthetized with 3% isoflurane (in mixed air and oxygen) and placed supine to expose the anterior cervical region. The right common carotid artery was separated from surrounding tissues and double ligated using 5-0 surgical silk suture. The artery was cut between the two ligations. The isoflurane exposure time was limited to 8 min. After surgery, pups were allowed to recover for 1 h and then placed in hypoxia (8% O_2_ and 92% N_2_), in an Erlenmeyer flask which was submerged in a 37 °C water bath, for 2.5 h. After hypoxia, all pups were returned to their dams. Sham pups underwent anesthesia and the exposure of the right common carotid artery, without the ligation and hypoxia.

### Intranasal administration

Pups were placed in a supine position under 2% isoflurane anesthesia at 1 h after HI. A total volume of 5 μL of STF083010 (45 μg/pup, Abcam) or vehicle (10% DMSO dissolved in corn oil) was administered intranasally. 1.25 μl of STF083010 or vehicle per drop was given every 2 min in alternating nares.

### Intracerebroventricular injection

The miR-125-b-2-3p inhibitor (0.5 nmol/pup, rno-miR-125b-22-3p miRCURY LNA miRNA Power Inhibitor, Qiagen, Cat#YI04109200-DDA), or the anti-miR control (0.5 nmol/pup, miRCURY LNA miRNA Power Inhibitor control, Qiagen, Cat#YI00199006-DDA) was administered to the right lateral ventricle at 24 h before HI. The intracerebroventricular injection was performed as previously described [30, 31]. Pups were placed in a stereotactic frame under isoflurane anesthesia. A 10-μl Hamilton micro syringe needle (Hamilton Company, USA) was inserted from the skull surface at the following coordinates relative to bregma: 1.5 mm posterior, 1.5 mm lateral to the bregma, and 1.7 mm beneath the horizontal plane of the skull. The miR-125-b-2-3p inhibitor or the anti-miR control was infused into the ventricle slowly over 5 min by a pump, and the needle was kept in place for 10 min after the end of each injection to prevent liquid reflux. The NLRP1 CRISPR activation plasmid (0.4 μg/pup, NLRP1 SAM guide RNA, Qiagen, Quote#U8376ED300) or control CRISPR activation plasmid (0.4 μg/pup, NLRP1 SAM guide RNA negative control, Qiagen, Quote#U8376ED300) was given via intracerebroventricular injection as described above at 24 h before HI. The time point for miRNA inhibitor and CRISPR injection was selected based on previous literature [32, 33]. According to the manufacturers’ protocol, the phenotypic effects of the products are normally assessed 24–72 h after delivery. A potentially attractive quality of antagomir targeting of miRNAs is prolonged suppression of the miRNA [34]. Silencing of miRNAs by antagomirs has been reported to last several weeks in the periphery [35] and after injection into the brain [36]. Therefore, we selected 24 h before HI as the best time point for intracerebroventricular injection.

### Experimental design

The experiment was designed as follows.

#### Experiment I

To study the temporal expression and cellular localization of p-IRE1α after hypoxia-ischemia (HI), rat pups were randomly divided into six groups: sham, HI-6 h, HI-12 h, HI-24 h, HI-48 h, HI-72 h. *n* = 6 per group. Western blot analysis was performed to determine the expression changes of IRE1α and p-IRE1α. Additional 6 pups in the HI-12 h group were used for double immunofluorescence staining to evaluate the co-localization of p-IRE1α with different cell types.

In addition, western blot analysis and reverse transcription quantitative real-time polymerase chain reaction (RT-qPCR) were performed to evaluate the temporal expression of NLRP1, cleaved caspase-1, IL-1β, IL-18, and miR-125-b-2-3p at various time points after HI (groups were assigned same to that of p-IRE1α time course detection).

#### Experiment II

To evaluate the neuroprotective effects of IRE1α inhibitor, STF083010, after HI, rat pups were randomly divided into 3 groups: sham, HI + vehicle (10% DMSO dissolved in corn oil), HI + STF083010. *n* = 18 per group, 6 pups for TTC staining and western blot, 6 pups for RT-qPCR, and 6 pups for immunofluorescence in each group. Western blot analysis, immunofluorescence, RT-qPCR, neurobehavioral tests, 2,3,5-Triphenyltetrazoliumchloride (TTC), and Fluoro-Jade C staining were performed to evaluate the infarct volume, neurological performance, the number of degenerating neurons, and the expression changes of miR-125-b-2-3p at 48 after HI with STF083010 treatment. In addition, double immunofluorescence staining was performed to determine the localization and expression changes of the inflammatory cytokines IL-1β and IL-18.

#### Experiment III

To explore whether miR-125-b-2-3p was involved in the underlying mechanisms of STF083010 mediated neuroprotective effects, antimiR-125 was used to inhibit miR-125-b-2-3p. Rat pups were randomly divided into 5 groups: sham, HI + vehicle, HI + STF083010, HI + STF083010 + antimiR control, HI + STF083010 + miR-125 inhibitor. *n* = 12 per group, 6 pups for TTC staining and western blot, 6 pups for RT-qPCR in each group. miR-125-b-2-3p inhibitor was intracerebroventricularly injected at 24 h before HI. TTC staining, neurobehavioral tests, western blot analysis, and RT-qPCR were examined at 48 h after HI.

#### Experiment IV

To explore whether NLRP1 was involved in the underlying mechanism of STF083010-mediated neuroprotective effects, NLRP1 activation CRISPR was used to activate NLRP1. Rat pups were randomly divided into 5 groups: sham, HI + vehicle, HI + STF083010, HI + STF083010 + CRISPR control, HI + STF083010 + NLRP1 CRISPR. *n* = 6 per group. NLRP1 activation CRISPR was intracerebroventricularly injected at 24 h before HI. TTC staining, neurobehavioral tests, and western blot analysis were examined at 48 h after HI.

### Neurobehavioral tests

Negative geotaxis test was performed to evaluate the neurological function at 48 after HI by two blinded investigators in an unbiased setup. The pups were placed head downward on a 45° inclined board, and the time taken for the pups to turn to head upward was recorded. The maximum testing time was 60 s, and the time taken more than 60 s was recorded as 60 s.

### Infarct volume measurement

At 48 h after HI, TTC staining was used to evaluate the infarct volume as previously described [30]. The rat pup brains were cut into 2-mm coronal sections in a rat brain matrix. The sections were incubated with 2% TTC solution (Sigma Aldrich Inc., USA) for 5 min at room temperature and then washed by phosphate-buffered saline (PBS). The sections were imaged, and the volume of the infarct area was quantified and analyzed with the Image J software (NIH, USA). The percentage of infarcted area for each section was calculated as [(total area of contralateral hemisphere) − (area of un-infarcted area of ipsilateral hemisphere)]/(total area of contralateral hemisphere × 2). The average value of each section in one brain was taken to represent the percentage of infarcted volume for that pup.

### Tissue processing

Pups were anesthetized with isoflurane and transcardially perfused with 4 °C PBS and 10% formalin. The brains were removed and post-fixed with formalin for 48 h and then immersed in 30% sucrose until they sank. After being embedded into OCT compound (Scigen Scientific) and frozen, the brains were sliced into serial 10-μm-thick coronal slices using a cryostat (CM3050S-3, Leica Microsystems) at − 20 °C. The brain slices were prepared for immunofluorescence and Fluoro-Jade C staining as follows.

#### Immunofluorescence staining

The slices were rinsed with PBS for 30 min and permeabilized with 0.3% Triton X-100 for 30 min at room temperature [37]. The slices were then rinsed with PBS for 15 min and blocked with 5% donkey serum at 37 °C for 30 min. Subsequently, each coronal slice was incubated with primary antibodies at 4 °C overnight. The primary antibodies used are as follows: rabbit anti-p-IRE1α (1:50, Abcam), mouse anti-NeuN (1:200, Santa Cruz Biotechnology), mouse anti-GFAP (1:100, Santa Cruze Biotechnology), and mouse anti-Iba-1(1: 200, Wako). Slices were rinsed in PBS for 15 min and then incubated with appropriate fluorescence-conjugated secondary antibodies at 37 °C for 1 h. After being rinsed in PBS for 15 min, the slices were covered with Vectashield Antifade Mounting Medium with DAPI (Vector Laboratories Inc.). Images were then visualized under a fluorescence microscope (Leica DMi8, Leica Microsystems).

#### Fluoro-Jade C staining

Fluoro-Jade C staining (FJC) was used to identify and quantify degenerating neurons. The staining was performed at 48 h post HI with the Fluoro-Jade C Ready-to-Dilute Staining Kit (Biosensis) following the manufacturer’s instructions. The number of FJC positive cells was counted manually in the peri-ischemic regions. The data was expressed as positive cells per mm^2^ and six sections per brain over a microscopic field of × 20 were picked to be averaged.

### Reverse transcription real-time quantitative polymerase chain reaction (RT q-PCR)

Total RNA extraction was isolated according to miRNeasy Mini Kit (Qiagen) instructions, and 2 μg of RNA in each group was reverse transcribed with miScript II RT kit (Qiagen) to generate cDNA. The expressions of miR-125-b-2-3p relative to SNORD61, an internal reference gene, were determined by SYBR-Green PCR method with miScript Primer Assay kit (Qiagen) according to manufacturer’s instructions. The primer of miR-125-b-2-3p was Rn-miR-125b*-2 miScript Primer Assay (Qiagen, Cat#MS000033201) and that of SNORD61 was Hs-SNORD61-11 miScript Primer Assay (Qiagen, Cat#MS00033705). The PCR reaction mixture consisted of 12.5 μl 2 × QuantiTect SYBR Green PCR Master Mix, 2.5 μl 10 × miScript Universal Primer, 2.5 μl 10 × miScript Primer Assay, 2 μl of template cDNA, and RNase-free water to a total volume of 25 μl. Cycling conditions were 95 °C for 15 min as an initial activation step, followed by 40 cycles: denaturation at 95 °C for 15 s, annealing at 55 °C for 30 s, and extension at 70 °C for 30 s. PCR specificity was confirmed by melt curve analysis. The relative fold change in miR-125-b-2-3p expression was calculated using the comparative cycle threshold method (2^−ΔΔCT^) [38].

### Western blot analysis

After TTC staining and digitally photographed at 48 h after HI, the brain sections were separated into contralateral and ipsilateral hemispheres immediately. The ipsilateral hemisphere samples were snap-frozen in liquid nitrogen and stored at − 80 °C for further use. The brain samples were homogenized in RIPA lysis buffer (Santa Cruz Biotechnology) with protease inhibitor cocktail for 15 min and then centrifuged at 14,000 *g* at 4 °C for 30 min, the whole cell lysates were collected. The protein concentration was determined by using a detergent compatibility assay (DC^TM^ Protein Assay, Bio-Rad). Equal amounts of proteins were loaded onto 10% sodium dodecylsulfate polyacrylamide gel electrophoresis (SDS-PAGE) gels for electrophoresis, and proteins were then transferred onto nitrocellulose membranes. The membranes were blocked for 2 h at 37 °C with 5% non-fat milk, followed by incubation at 4 °C overnight with the primary antibodies: anti-p-IRE1α (1:500, Abcam), anti-IRE1α (1:500, Abcam), anti-NLRP1 (1:1000, Abcam), anti-cleaved caspase-1 (1:500, Novus Biologicals), anti-IL-1β (1:1000, Abcam), anti-IL-18 (1:1000, Abcam), and anti-β-actin (1:3000, Santa Cruz Biotechnology). The membranes were then incubated for 1 h at 37 °C with horseradish peroxidase-conjugated secondary antibodies (Santa Cruz Biotechnology). Bands were visualized using ECL Plus kit (American Bioscience, UK) and quantified through the Image J software. The density of each protein of interest was normalized against the density of the β-actin band.

### Statistical analysis

Statistical analysis was performed using the GraphPad Prism 7.01 software. The data were presented as mean ± SD. Differences between individual groups were determined with one-way ANOVA analysis of variance followed by post hoc tests with Tukey’s multiple comparisons. Differences between two groups were compared using Student’s *t* test. All reported *P* values were two-sided, and a value of *P* < 0.05 was considered statistically significant [39].

## Results

### Spatial expression and time course of endogenous p-IRE1α expression after HI

**Fig. 1:**
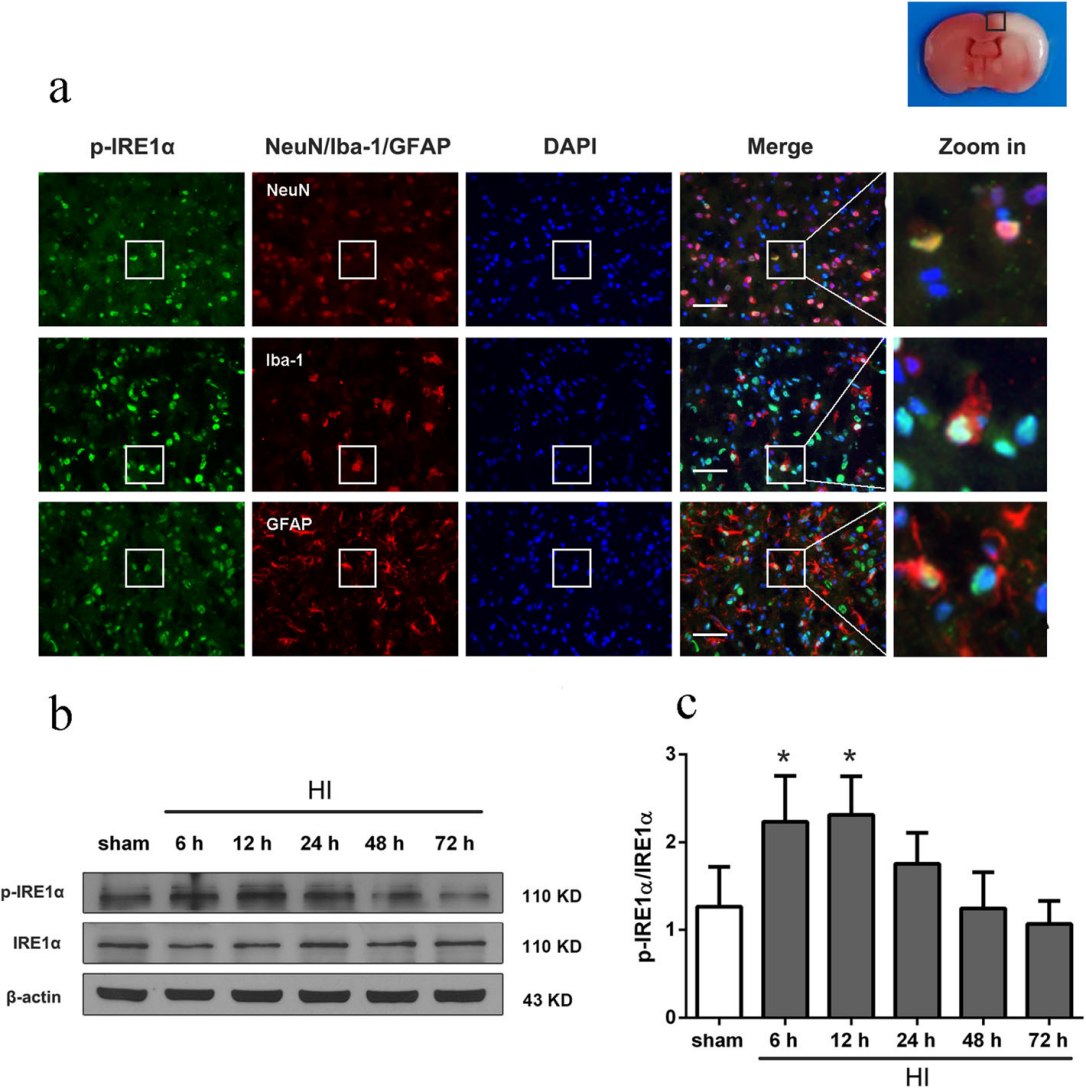
Endogenous expression of p-IRE1α after HI insult. **a** Double immunostaining of p-IRE1α with neurons (NeuN), astrocytes (GFAP), and microglia (Iba-1) at 12 h after HI. *n* = 6 per group. Top panel indicates the location of microphotographs (small black box). Scale bar = 50 μm. **b**, **c** Representative western blot bands of time course, and quantitative analysis of the relative expression levels of p-IRE1α /IRE1α after HI. *n* = 6 per group, ^*^P < 0.05 *vs.* sham group

Double immunofluorescence staining of p-IRE1α with NeuN, Iba-1, and GFAP was performed at 12 h post HI. Colocalization of p-IRE1α with NeuN, Iba-1, and GFAP were detected in ipsilateral peri-infarct cortex (Fig. 1a). These results indicated that IRE1α was extensively expressed on neurons, microglia, and astrocytes. Analysis of the western blot bands showed that the ratio of p-IRE1α/IRE1α expression increased in a time-dependent manner, reaching peak at 12 h after HI (*P* < 0.05 compared to sham group, Fig. 1b, c).

### Expression changes of NLRP1, cleaved caspase-1, IL-1β, IL-18, and miR-125-b-2-3p after HI

**Fig. 2:**
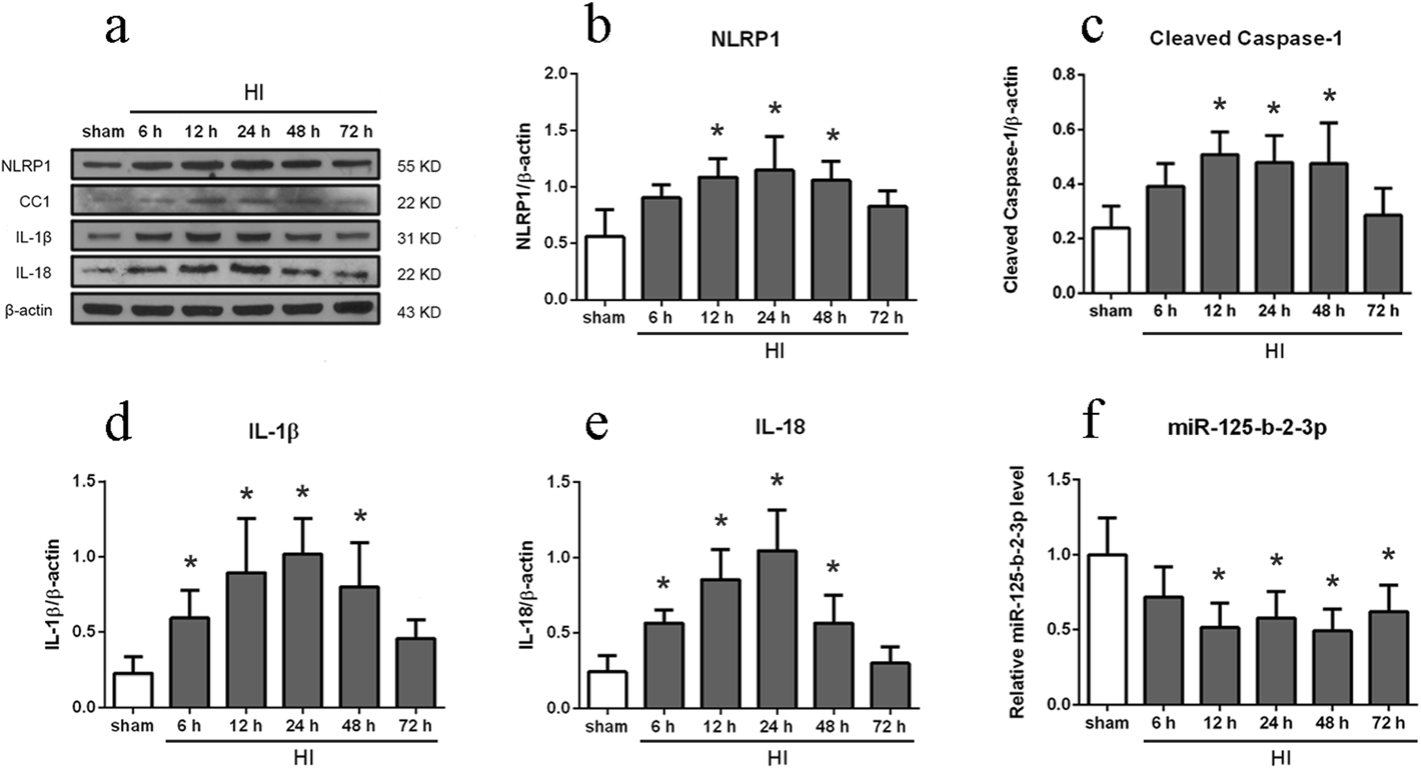
Endogenous expression of NLRP1, cleaved caspase-1, IL-1β, IL-18, and miR-125-b-2-3p after HI insult. **a**–**e** Representative western blot bands of time course, and quantitative analysis of the relative expression level of proteins NLRP1, cleaved caspase-1 (CC1), IL-1β, and IL-18 after HI insult, *n* = 6 per group. **f** Expression of endogenous miR-125-b-2-3p after HI insult determined by real time PCR detection, *n* = 6 per group. ^*^P < 0.05 *vs.* sham group

Analysis results of the western blot bands showed the expression level of NLRP1, cleaved caspase-1, IL-1β, and IL-18 dramatically increased after HI induction and reached to the highest level at 12 h or 24 h post HI (*P* < 0.05 compared to sham group, Fig. 2a–e). These results indicated that the NLRP1 inflammasome was activated and neuronal cell pyroptosis occurred in the HIE rat model.

RT-qPCR results showed that the expression level of miR-125-b-2-3p dramatically decreased after HI induction and reached to the lowest level at 48 h post HI (*P* < 0.05 compared to sham group, Fig. 2f).

### Intranasal administration of STF083010 reduced infarct volume, improved short-term neurological functions, reduced neuronal degeneration, and upregulated miR-125-b-2-3p expression at 48 h after HI

**Fig. 3:**
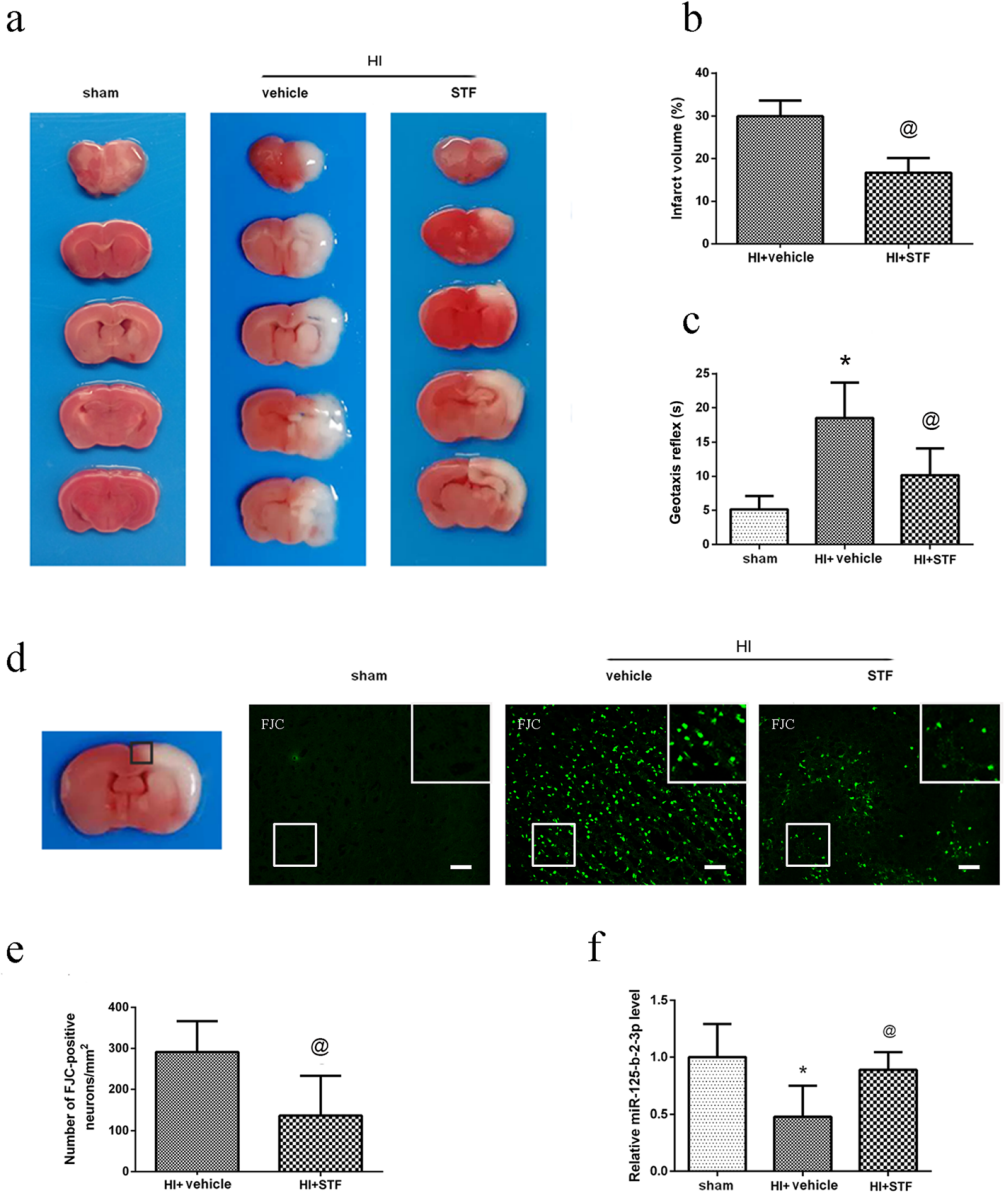
Effect of intranasal administration of STF083010 on brain infarct volume, neuronal degeneration, neurological function and the relative miR-125-b-2-3p expression level. **a** Representative TTC staining photographs. **b** Analysis of infarct volume. **c** Analysis of reflex time in Geotaxis test. **d** Representative Fluoro-Jade C staining (FJC) photographs. Left panel indicates the location of microphotographs (small black box). The white box at right upper corner in each FJC staining photographs is the magnification of that at left lower corner. Scale bar = 100 μm. **e** Analysis of the number of FJC-positive neurons in the FJC staining photographs. **f** Quantitative analysis of the relative expression level of miR-125b-2-3p in real time PCR tests. Data are represented as means ± SD. *n* = 6 for each group. ^*^P < 0.05 *vs.* sham group; ^@^*P* < 0.05 *vs.* HI + vehicle group

TTC staining results showed that there was no infarct region in sham group. HI insults lead to the obvious infarct area in vehicle group, while intranasal administration of STF083010 reduced the infarct volume when compared with vehicle group (*P* < 0.05, Fig. 3a, b).

The Geotaxis reflex time in vehicle group was increased significantly when compared with sham group at 48 h after HI, while it decreased significantly in STF083010 treatment group when compared with vehicle group (*P* < 0.05, Fig. 3c).

Robust FJC staining in ipsilateral peri-infarct cortex was observed at 48 h after HI in vehicle group, while STF083010 treatment significantly reduced the number of FJC-positive neurons when compared with vehicle group (*P* < 0.05, Fig. 3d, e).

RT q-PCR data showed that the expression of miR-125-b-2-3p significantly decreased in vehicle group when compared with sham group (*P* < 0.05), while STF083010 treatment upregulated the expression of miR-125-b-2-3p when compared with vehicle group (*P* < 0.05, Fig. 3f).

These results indicated that IRE1-α inhibition reduced infarct volume, improved short-term neurological function, reduced neuronal degeneration, and upregulated miR-125-b-2-3p expression after HI.

### Intranasal administration of STF083010 downregulated the expression level of IL-1β and IL-18 at 48 h after HI

**Fig. 4:**
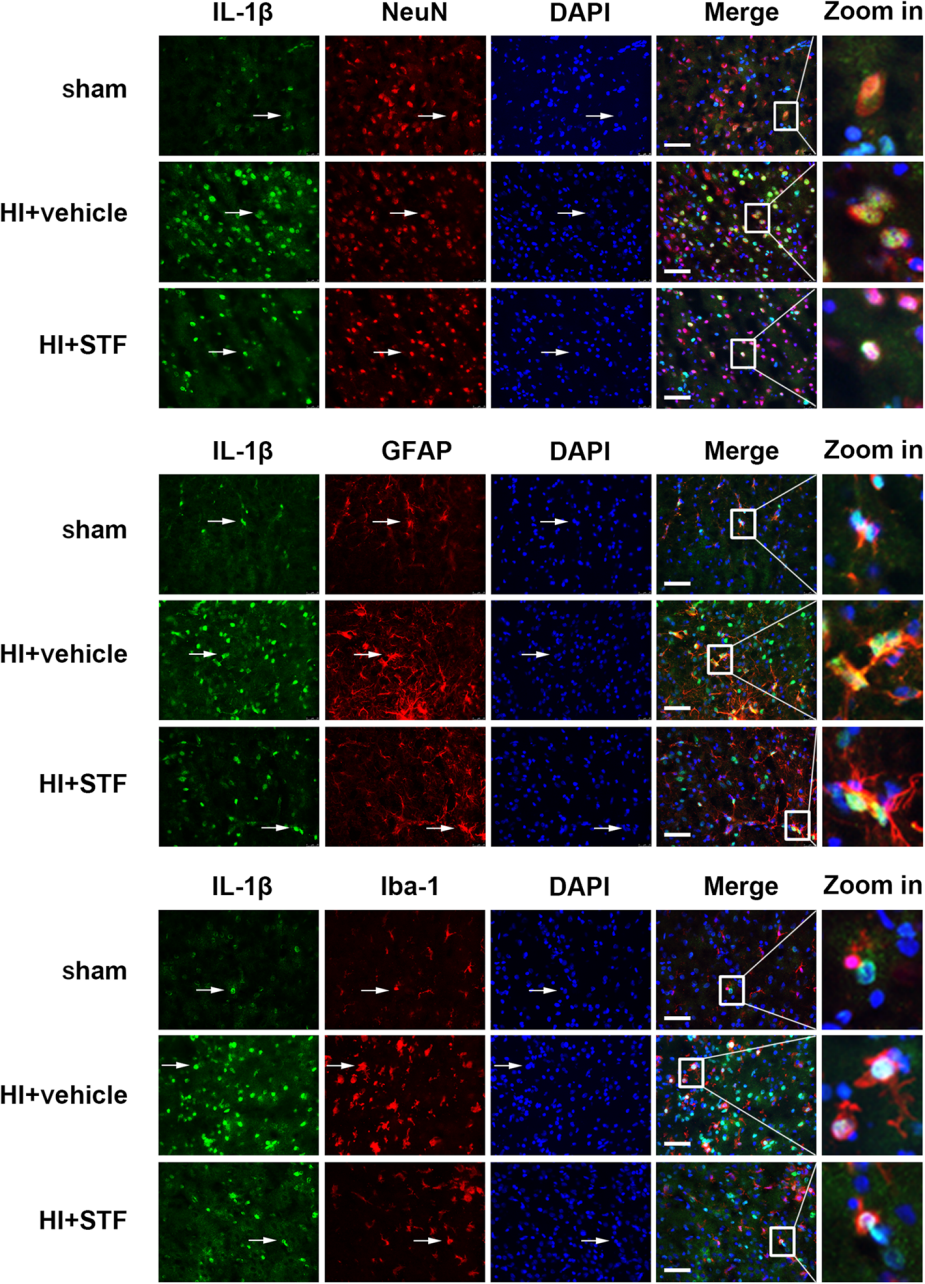
Representative immunofluorescence microphotographs of IL-1β co-expressed with NeuN, GFAP, and Iba-1 respectively in sham, HI + vehicle, and HI + STF083010 groups. Top right panel indicates the location of microphotographs (small black box). Scale bar = 50 μm. *n* = 6 for each group

**Fig. 5:**
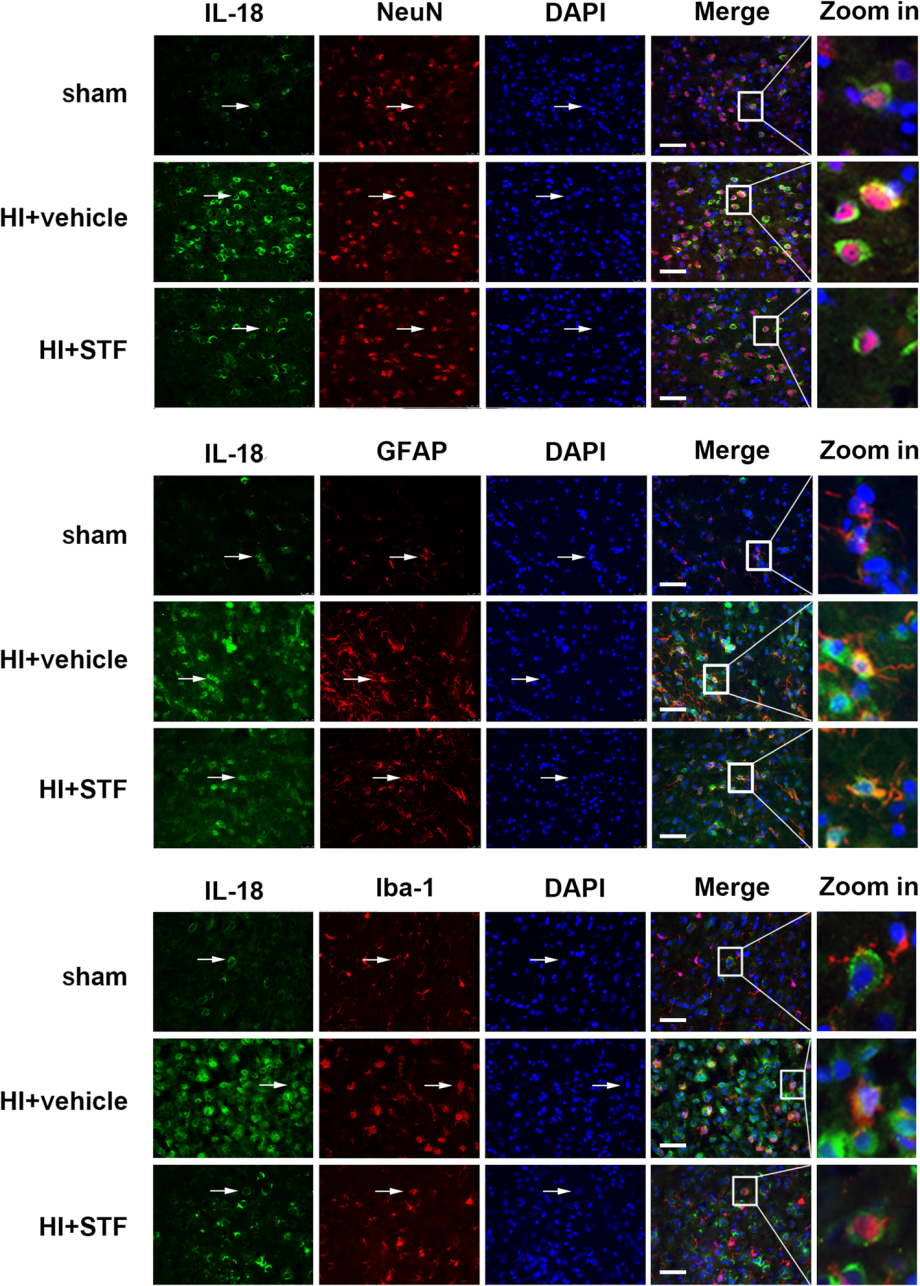
Representative immunofluorescence microphotographs of IL-18 co-expressed with NeuN, GFAP, and Iba-1 respectively in sham, HI + vehicle, and HI + STF083010 groups. Top right panel indicates the location of microphotographs (small black box). Scale bar = 50 μm. *n* = 6 for each group

Immunofluorescence staining showed that the fluorescent intensity of IL-1β and IL-18 on neurons, astrocytes, and microglia, in vehicle group, was increased when compared with sham group, while the fluorescent intensity of the two inflammatory cytokines decreased in STF083010 treatment group when compared with the vehicle group. IL-1β and IL-18 were expressed extensively on neurons, microglia, and astrocytes (Fig. 4, Fig. 5). These results indicated that IRE1-α inhibition reduced cell pyroptosis after HI.

### Inhibition of miR-125-b-2-3p attenuated the neuroprotective effects induced by STF083010 treatment at 48 h post HI

**Fig. 6:**
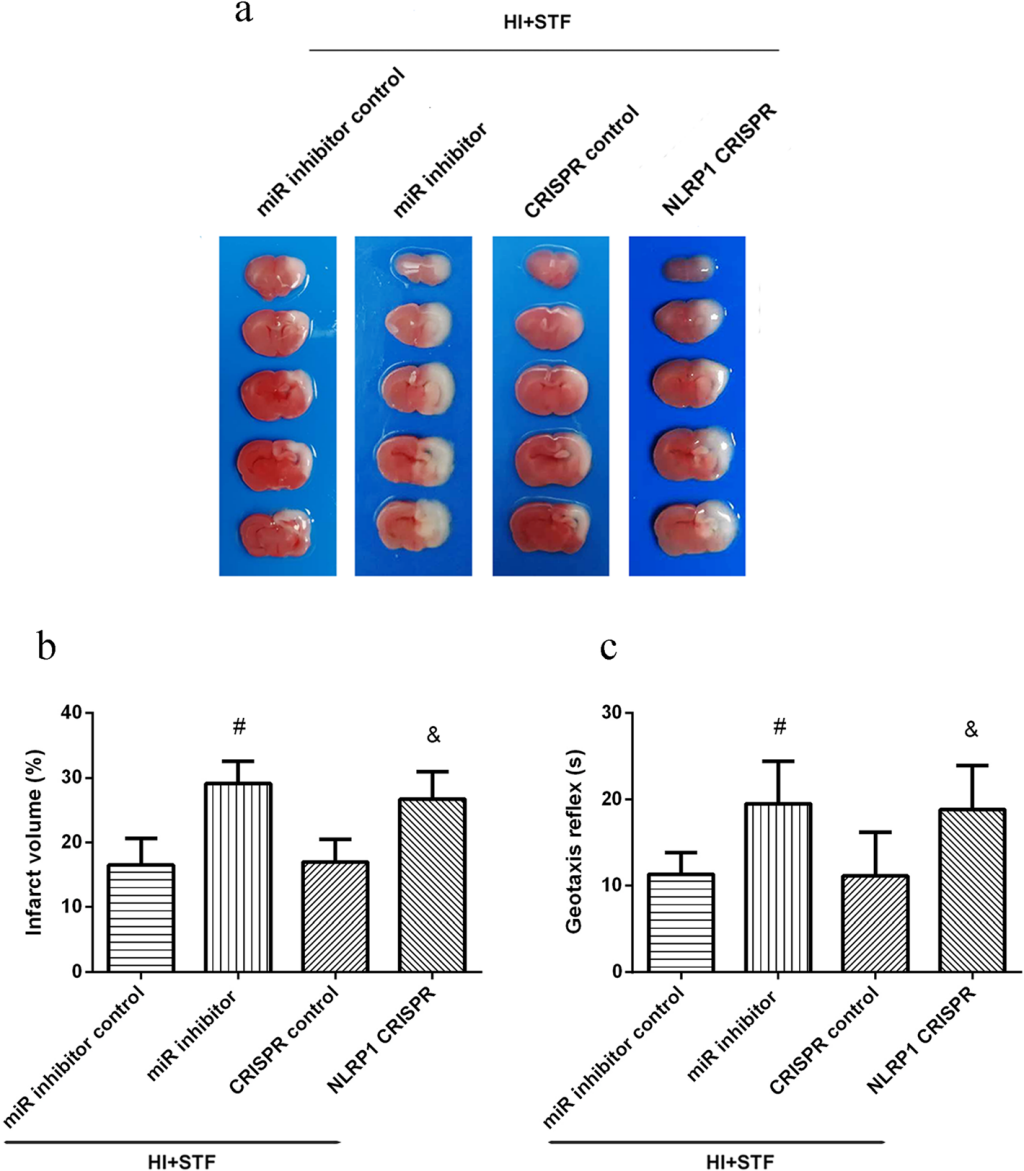
Effects of miR-125 inhibitor and NLRP1 CRISPR on infarct volume and neurological function at 48 h post HI. **a** Representative TTC staining photographs. **b** Analysis of infarct volume. **c** Analysis of reflex time in Geotaxis test. Data are represented as means ± SD. *n* = 6 for each group. ^#^*P* < 0.05 *vs*. HI + STF083010 + miR inhibitor control group; ^&^*P* < 0.05 *vs.* HI + STF083010 + CRISPR control

To determine the role of miR-125-b-2-3p in STF083010 neuroprotective effects, we used antimiR-125 to inhibit miR-125-b-2-3p. TTC staining results showed that the infarct volume increased significantly in HI + STF083010 + antimiR-125 group when compared with the HI + STF083010 + miR inhibitor control group. The Geotaxis reflex time increased in HI + STF083010 + antimiR-125 group when compared with the HI + STF083010 + miR inhibitor control group (*P* < 0.05, Fig. 6). These results indicated that miR-125-b-2-3p was involved in the neuroprotective effects induced by IRE1-α inhibition.

### Inhibition of miR-125-b-2-3p reversed the downregulation of NLRP1, cleaved caspase-1, IL-1β, and IL-18 induced by STF083010 treatment at 48 h post HI

**Fig. 7:**
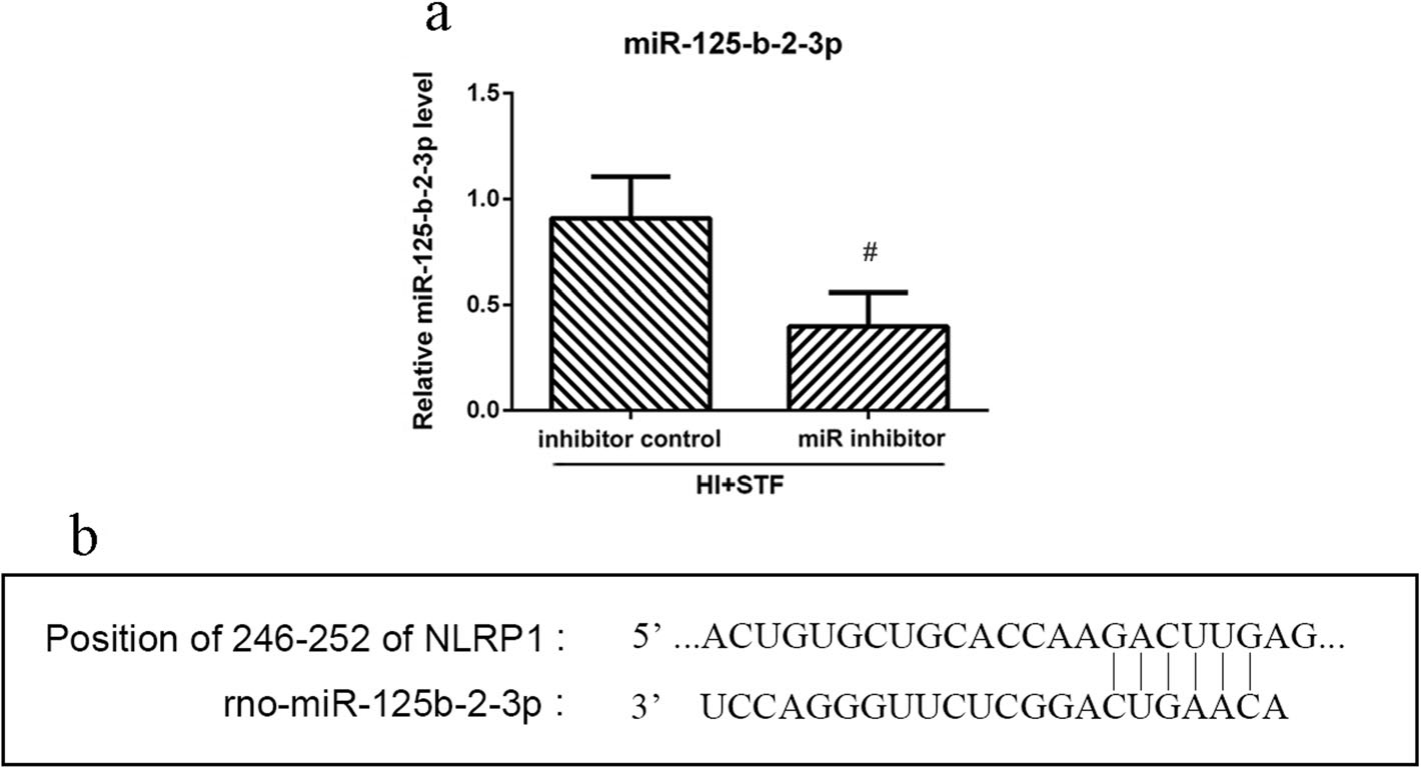
Effects of miR-125 inhibitor on miR-125b-2-3p and search results from the TargetScan software. **a** Quantitative analysis of the relative expression level of miR-125b-2-3p in real time PCR tests. Data are represented as means ± SD. *n* = 6 for each group. ^#^*P* < 0.05 *vs.* HI+ STF083010 + miR inhibitor control. **b** Sequence alignment showed putative miR-125b-2-3p binding sites within the 3’-UTR of the NLRP1 mRNA in rats (http://www.targetscan.org/)

RT q-PCR data showed the expression level of miR-125-b-2-3p decreased significantly in HI + STF083010 + antimiR-125 group when compared with the HI + STF083010 + miR inhibitor control group (*P* < 0.05, Fig. 7a).

**Fig. 8:**
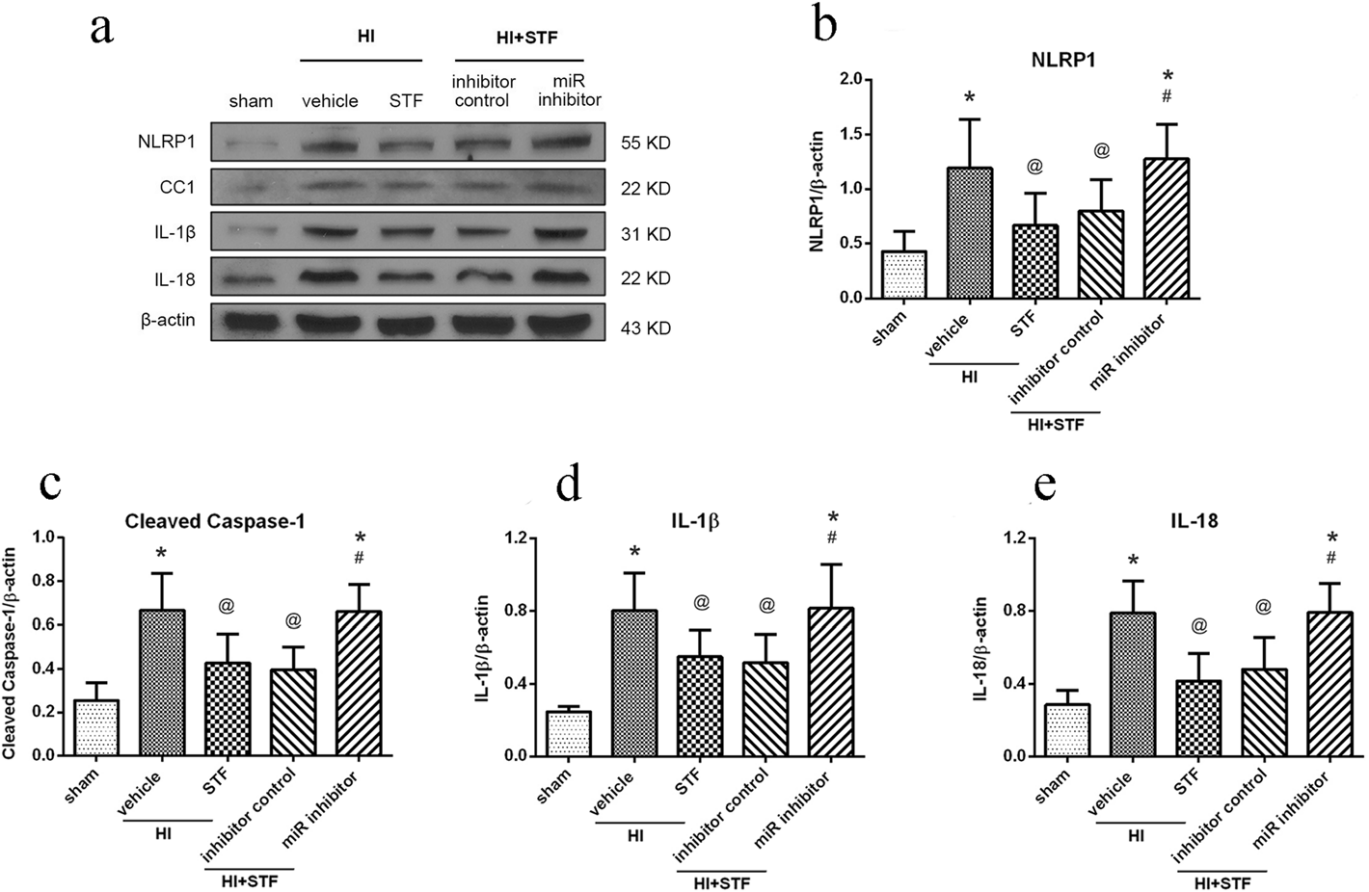
Effects of miR-125 inhibitor on downstream proteins in proposed signaling pathway with STF083010 treatment at 48 h post HI. **a** Representative pictures of the western blot bands of the proteins NLRP1, cleaved caspase-1(CC1), IL-1β, and IL-18. **b**–**e** Quantitative analysis of the relative expression level of proteins NLRP1, cleaved caspase-1, IL-1β, and IL-18. Data are represented as means ± SD. *n* = 6 for each group. ^*^P < 0.05 *vs.* sham group; ^@^*P* < 0.05 *vs.* HI + Vehicle; ^#^*P* < 0.05 *vs.* HI + STF083010 + miR inhibitor control

Western blot data showed that the expression level of NLRP1, cleaved caspase-1, IL-1β, and IL-18 increased significantly in HI + vehicle group when compared with the sham group, and then decreased significantly in HI + STF083010 group when compared with the HI + vehicle group. These proteins increased significantly in HI + STF083010 + antimiR-125 group when compared with the HI + STF083010 + miR inhibitor control group (*P* < 0.05, Fig. 8).

These results indicated that miR-125-b-2-3p plays an important protective role against ER stress-induced cell pyroptosis.

### NLRP1 activation CRISPR attenuated the neuroprotective effects induced by STF083010 treatment at 48 h post HI

To explore whether NLRP1 was involved in the mechanism of STF083010 neuroprotective effects, we used NLRP1 activation CRISPR to activate NLRP1 expression. TTC staining results showed the infarct volume increased significantly in HI + STF083010 + NLRP1 activation CRISPR group when compared with the HI + STF083010 + CRISPR control group. The Geotaxis reflex time increased in HI + STF083010 + NLRP1 activation CRISPR group when compared with the HI + STF083010 + CRISPR control group (*P* < 0.05, Fig. 6). These results indicated that NLRP1 inflammasome was involved in the neurological injury induced by IRE-1α activation.

### NLRP1 activation CRISPR reversed the downregulation of NLRP1, cleaved caspase-1, IL-1β, and IL-18 induced by STF083010 treatment at 48 h post HI

**Fig. 9:**
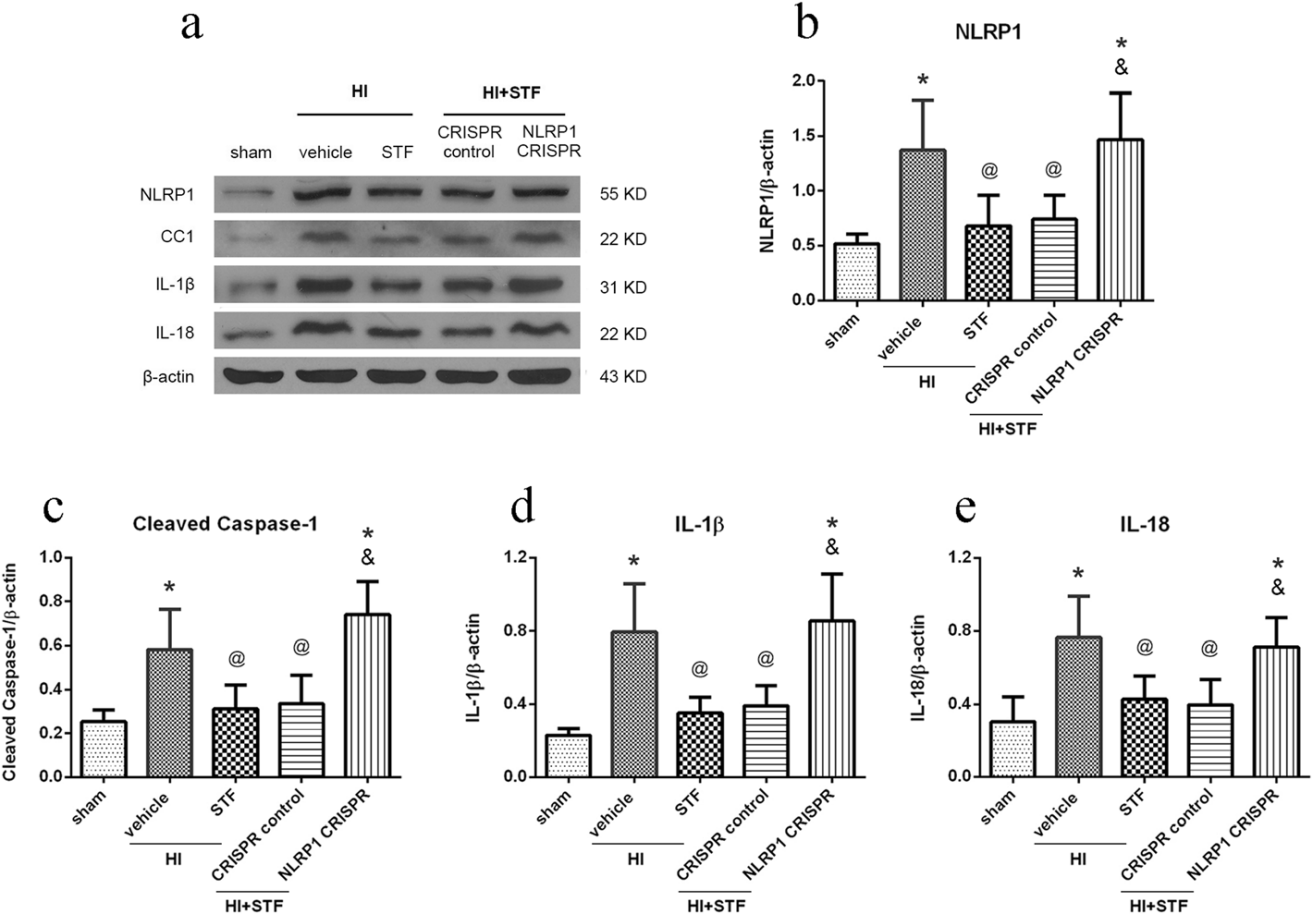
Effects of NLRP1 activation CRISPR on downstream proteins in proposed signaling pathway with STF083010 treatment at 48 h post HI. **a** Representative pictures of the western blot bands of the proteins NLRP1, cleaved caspase-1(CC1), IL-1β, and IL-18. **b**–**e** Quantitative analysis of the relative expression level of proteins NLRP1, cleaved caspase-1, IL-1β, and IL-18. Data are represented as means ± SD. *n* = 6 for each group. *P < 0.05 *vs.* sham group; ^@^*P* < 0.05 *vs.* HI + Vehicle; ^&^*P* < 0.05 *vs.* HI + STF083010 + CRISPR control

Western blot data showed that the expression level of NLRP1, cleaved caspase-1, IL-1β, and IL-18 increased significantly in HI + vehicle group when compared with the sham group, and then decreased significantly in HI + STF083010 group when compared with the HI + vehicle group. These proteins increased significantly in HI + STF083010 + NLRP1 activation CRISPR group when compared with the HI + STF083010 + CRISPR control group (*P* < 0.05, Fig. 9). These results indicated that NLRP1 inflammasome was involved in ER stress-induced cell pyroptosis after HI.

## Discussion

The activation of several different neuronal cell death pathways, including apoptosis, autophage, pyroptosis, necrosis, excitotoxic cell death, etc., account for the brain damages that occur after hypoxia-ischemic (HI) insults [40–43]. Recently, the contribution of neuronal pyroptosis in the process of HIE pathophysiology is drawing much more attention and many researchers have tried to reduce neural damage by reversing the occurrence of neuronal pyroptosis in HIE [41, 44, 45]. NLRP1 was the first inflammasome to be characterized and was reported to promote pyroptosis in neurons [22, 46]. The core complex of NLRP1 inflammasome is composed of NLRP1, ASC (apoptosis-associated speck-like protein containing a CARD), caspase 1, and caspase 5 [22]. In the present study, western blot results showed that the expression level of NLRP1, cleaved caspase-1, IL-1β, and IL-18 were increased after HI insult, which indicated that NLRP1 inflammasome was activated and neuronal cell pyroptosis occurred in the HIE rat model. The immunofluorescence results indicated that both inflammatory cytokines, IL-1β and IL-18, were expressed in neurons, microglia, and astrocytes, demonstrating pyroptosis.

ER stress, caused by the clustering of excess misfolded or unfolded proteins in the ER lumen, triggers unfolded protein response (UPR) signaling pathway [47]. Under remediable ER stress, UPR is a protective response aimed at preventing further accumulation of unfolded proteins within the ER and returning the ER to its normal physiological state. However, under severe or prolonged ER stress, UPR switches to leading cell death by activating ER-associated apoptotic pathways [4, 5, 48, 49]. At least three ER-transmembrane proteins, the PKR-like ER kinase (PERK), the activating transcription factor-6 (ATF-6), and the inositol-requiring enzyme 1 (IRE1), contain ER luminal stress-sensing domains and carry out the UPR in mammals. IRE1 is the most ancient, evolutionarily conserved UPR sensor, which has two types of isoforms: IRE1α or IRE1β [11, 50, 51]. IRE1α is expressed extensively in various tissues, whereas IRE1β is only expressed in the digestive system [11, 50]. Apart from the stress sensor domain, IRE1α contains a cytosolic serine/threonine kinase domain and an endoribonuclease (RNase) domain as well [47, 52, 53]. Upon ER stress, the IRE1α kinase domain is dimerized and self-phosphorylated, which consequently activates the RNase domain [47, 54, 55]. The RNase activity of IRE1α catalyzes the excision of a 26-nt intron within the X-box-binding protein 1 (XBP1) mRNA and results in the formation of spliced XBP1 (XBP1s), which transcriptionally promotes the expression of genes responsible for restoring ER folding capacity [56–58]. In addition to its adaptive effects on alleviating unfolded protein accumulation, excessive activation of IRE1α can switch to promote apoptosis by degrading numerous types of RNA which encode the downstream signal molecules related to apoptosis such as CCAAT/enhancer-binding protein-homologous protein (CHOP) [47, 59, 60]. In the present study, the results showed that the relative expression level of p-IRE1ɑ peaked at 6 h and started to decline after 12 h, which is consistent with previous studies [4, 5] and indicated that IRE1ɑ activation, after HIE, is an early event.

In our previous study, we explored the optimal dose of STF083010 (45 μg/pup), a specific inhibitor of IRE1α’s RNAase activity, for treatment in HIE rat model and evaluated STF083010 protective effects by measuring infarct volume and performing neurobehavioral testing at 24 h and 72 h after HI insult [5]. Here we complementally observed the treatment effect of the best dose of STF083010 on infarct volume and negative geotaxis test score at 48 h after HI insult, which further confirmed that inhibition of the IRE1α activity could alleviate the brain injury in HIE rat model.

Severe ER stress is associated with multiple types of tissue damage, including excitotoxicity injury, oxidative stress, and neuronal apoptosis [61–63]. For example, the accumulation of excessive unfolded proteins within the ER leads to the hyperfusion of mitochondria and initiates mitochondria-mediated apoptosis in cells [64]. It was founded that IRE1α inhibition reduced the number of FJC-positive neurons in the present study, which indicates that severe ER stress is responsible for the degeneration of neurons in HIE rat model. The factors accounting for the degeneration of neurons include neuronal apoptosis, necrosis, pyroptosis, etc. [65–67]. The type of cell death, which associates ER stress with neurodegeneration, is an issue that needs in-depth investigation. Here, we focused on the relationship between ER stress process with neuronal pyroptosis and found that inhibition of IRE1α leads to the decreased expression of cleaved caspase-1, IL-1β, and IL-18. These results indicate that ER stress could initiate neuronal injury through inducing neuronal pyroptosis in HIE rat model. In our study, we demonstrated ER stress-induced neuronal pyroptosis; however, the role of other ER stress-induced signaling pathways which leads to the brain injury through oxidative stress or excitotoxicity needs to be further evaluated.

The cytosolic pattern-recognition receptors that form the inflammasomes in the central nervous system (CNS) are either a member of the PYHIN (pyrin and HIN domain-containing) family of proteins or a member of the NLRs (NOD-like receptors) family proteins [22]. The members of PYHIN family proteins consists of absent in melanoma 2 (AIM2) and interferon-inducible protein 16(IFI16), and those of NLRs family are comprised of NLRP1 (NOD-, LRR- and pyrin domain-containing 1), NLRP2, NLRP3, NLRP6, NLRP12, and NLRC4 (activation and recruitment domain –containing 4) [22]. Among the above numerous inflammasomes, NLRP1 and NLRP3 were extensively studied. In our previous study, we showed that destabilizing the NLRP3/TXNIP inflammasome led to neuroprotection after HIE [5]. In our present study, we demonstrated that inhibition of IRE1α downregulated the expression of NLRP1 in HIE model. Moreover, NLRP1 activation CRISPR reversed STF083010s effects by upregulating the expressions of cleaved caspase-1, IL-1β, and IL-18 after HI insult. These results suggest that NLRP1 inflammasome may contribute to neuronal pyroptosis induced by IRE1α activation after HIE. In addition to the activation of IRE1α, there are two other transmembrane sensor proteins, PERK and ATF-6, that can be activated as well in the course of the UPR. Whether activation of the other two UPR sensor proteins, PERK and ATF-6, also lead to the downstream pathways related to the formation of cytosolic inflammasomes and neuronal pyroptosis is worth of study in the future.

Recent studies have shown that miR-125 plays important roles in regulating cancer proliferation, invasion, angiogenesis, liver regeneration, etc. [68–71]. In this study, the expression of p-IRE1α was increased and miR-125 was decreased in HIE rat model; while the expression of miR-125 was upregulated after inhibition of IRE-1α activity. These results suggest that the activation of IRE-1α promotes the degradation of miR-125, which is consistent with previous reports [13]. Considering NLRP1 being upregulated after miR-125 was inhibited, and that a nucleotide sequence in the NLRP1 3’ UTR might be targeted by miR-125 in the search results of TargetScan software, we drew the conclusion that miR-125 acts as a bridge between the RNase activity of IRE1α and the stability of NLRP1 inflammasome. The decay of miR-125 caused by IRE1α activation in HIE model leads to the occurrence of neuronal pyroptosis through promoting the formation of NLRP1 inflammasomes.

## Conclusions

In conclusion, this study demonstrated that inhibition of the excessive RNase activity of IRE1α is protective in part via the miR-125/NLRP1 signaling pathway in neonatal HIE rat model, and IRE1α inhibitor acts to reverse the neuronal pyroptosis post HIE. Elucidating extensively the signaling pathway involved in neuronal pyroptosis in the UPR process is important for future novel treatment targets to reduce neuronal injury caused by irreversible ER stress post HIE.

## Data Availability

The data used in this study are available from the corresponding author on reasonable request.

## Abbreviations

AIM2: Absent in melanoma 2
ATF-6: Activating transcription factor-6
CC1: Cleaved caspase-1
CHOP: CCAAT/enhancer-binding protein-homologous protein
CNS: Central nervous system
ER: Endoplasmic reticulum
FJC: Fluoro-Jade C
GFAP: glial fibrillary acidic protein
HI: Hypoxia-ischemia
HIE: Hypoxic-ischemic encephalopathy
Iba-1: Ionized calcium binding adaptor molecule-1
IFI16: Interferon-inducible protein 16
IL: Interleukin
IRE1α: Inositol requiring enzyme-1 alpha
miRNAs: MicroRNAs
miR-125-b-2-3p: MicroRNA-125-b-2-3p
NeuN: Neuronal nuclei
NLRP1: NOD-, LRR- and pyrin domain-containing 1
NOD: Nucleotide-binding oligomerization domain
PERK: PKR-like ER kinase
p-IRE1α: Phosphorylated inositol requiring enzyme-1 alpha
PYHIN: Pyrin and HIN domain-containing
RNase: Endoribonuclease
RT-qPCR: Reverse transcription quantitative real-time polymerase chain reaction
SDS-PAGE: Sodium dodecylsulfate polyacrylamide gel electrophoresis
TTC: 2,3,5-Triphenyltetrazoliumchloride
UPR: Unfolded protein response
XBP1: X-box-binding protein 1
XBP1s: Spliced XBP1
